# A Transient Inflammatory Response Induced by Lipopolysaccharide Infusion Lowers Markers of Endogenous Cholesterol and Bile Acid Synthesis in Healthy Normocholesterolemic Young Men

**DOI:** 10.3390/biomedicines11010126

**Published:** 2023-01-04

**Authors:** Sultan Mashnafi, Sabine Baumgartner, Ronald P. Mensink, Desiree Perlee, Lonneke A. van Vught, Dieter Lütjohann, Jogchum Plat

**Affiliations:** 1 Department of Nutrition and Movement Sciences, NUTRIM School of Nutrition and Translational Research in Metabolism, Maastricht University Medical Center, 6200 MD Maastricht, The Netherlands; 2 Department of Medical Basic Sciences, Faculty of Applied Medical Sciences, AlBaha University, AlBaha 65779-7738, Saudi Arabia; 3 Center for Experimental and Molecular Medicine, Academic Medical Center, University of Amsterdam, 1105 AZ Amsterdam, The Netherlands; 4 Institute of Clinical Chemistry and Clinical Pharmacology, University Hospital Bonn, Venusberg-Campus 1, 53127 Bonn, Germany

**Keywords:** LPS-induced inflammation, cholesterol absorption, cholesterol synthesis, bile acid synthesis, non-cholesterol sterols

## Abstract

Inflammation is associated with changes in plasma lipids, lipoproteins, and cholesterol efflux capacity (CEC). It is unknown if the changes in lipids and lipoproteins during inflammation are related to changes in cholesterol absorption, synthesis, and bile acid synthesis. We, therefore, examined the effects of acute lipopolysaccharide (LPS)-induced transient systemic inflammation on lipids, lipoproteins, CEC, and markers of cholesterol metabolism. We also evaluated whether markers for cholesterol metabolism at baseline predict the intensity of the inflammatory response. Eight healthy young subjects received LPS infusion, and blood was sampled for the following 24 h. In addition to lipids, lipoproteins, and CEC, we also measured markers for cholesterol absorption and synthesis, bile acid synthesis, and inflammation. Compared with baseline, plasma total cholesterol, low-density lipoprotein cholesterol, and CEC decreased, while triglycerides increased in the 24 h following LPS infusion. TC-standardized levels of cholesterol synthesis markers (lathosterol, lanosterol, and desmosterol) and a bile acid synthesis marker (7α-OH-cholesterol) also decreased, with no changes in cholesterol absorption markers (campesterol, sitosterol, and cholestanol). Baseline TC-standardized levels of desmosterol and 7α-OH-cholesterol were positively correlated with concentrations of various inflammatory markers. Changes in TC-standardized desmosterol and 7α-OH-cholesterol were negatively correlated with concentrations of inflammatory markers. LPS infusion reduced endogenous cholesterol synthesis and bile acid synthesis in healthy young men.

## 1. Introduction

Atherosclerosis is a process underlying the development of cardiovascular diseases (CVDs), which are the leading cause of mortality worldwide [1]. Lipid abnormalities are a well-known risk marker for the development of atherosclerosis [2]. However, evidence highlighting the importance of inflammation in the initiation and progression of atherosclerosis is expanding rapidly [3]. For example, lowering inflammation by targeting interleukin-1beta (IL-1β) reduced the occurrence of CVD events, even when lipid profiles were not affected [4]. In addition to the direct effects of inflammation on the vasculature [5], there are also clear indications that inflammation may affect serum total cholesterol (TC), high-density lipoprotein cholesterol (HDL-C), low-density lipoprotein cholesterol (LDL-C), and triglyceride (TG) concentrations [6,7]. Moreover, alterations in not only circulating levels of HDL but also HDL composition, size, and functionality have been observed during inflammation [8,9,10], but the studies are not conclusive [11]. Although these associations between inflammation and circulating lipoproteins are now acknowledged, it is unclear whether changes in plasma lipid and lipoprotein concentrations and decreases in HDL functionality during inflammation are related to changes in intestinal cholesterol absorption, endogenous cholesterol synthesis, and/or bile acid synthesis, which are main processes regulating cholesterol homeostasis. However, the etiologies of inflammatory diseases are very different, which makes it difficult to compare studies. Therefore, infusion of lipopolysaccharide (LPS), a toxin derived from Gram-negative bacteria, has been proposed as a controlled experimental setting and model to induce a transient acute phase response without the presence of an infection [12,13]. Indeed, a clear inflammatory response and endothelial cell activation upon LPS infusion have frequently been observed [13]. Others have shown changes in serum lipid and lipoproteins concentrations such as reductions in TC and increases in TG after LPS administration in humans [6,14]. In addition, inflammation was associated with alterations in HDL composition and size and with decreased functionality following 24 h of LPS infusion [15], while the effects of LPS-induced inflammation on key processes regulating cholesterol homeostasis are unknown.

Analyzing intestinal cholesterol absorption, endogenous cholesterol synthesis, and bile acid synthesis usually requires the laborious stable isotope tracer methodology [16,17,18]. However, plasma non-cholesterol sterols are frequently used as markers for evaluating changes in cholesterol metabolism. The cholesterol precursors desmosterol and lathosterol reflect endogenous cholesterol synthesis, while the non-cholesterol sterols sitosterol, campesterol, and cholestanol reflect fractional intestinal cholesterol absorption [19]. Finally, 7α-OH-cholesterol and 27-OH-cholesterol can be used as markers for bile acid formation [20]. Interestingly, some of these non-cholesterol sterols might also affect inflammatory responses, i.e., for the cholesterol precursor desmosterol as well as for several oxysterols, anti-inflammatory effects have been described via activating liver X receptors (LXR) [21]. Therefore, we decided to evaluate the effects of acute LPS-induced transient systemic inflammation on plasma lipid and lipoprotein concentrations, HDL functionality, and markers reflecting cholesterol metabolism (absorption, synthesis, and bile acid formation). In addition, it was examined whether the baseline characteristics of these markers were able to predict the LPS-induced transient inflammatory response.

## 2. Materials and Methods

### 2.1. Subjects and Study Design

This study had a randomized, placebo-controlled, single-blind, parallel design and was carried out at the Center of Experimental and Molecular Medicine, Academic Medical Center, University of Amsterdam, The Netherlands. Details of this study have been described elsewhere [13]. Briefly, 32 healthy men were divided into 4 treatment arms: infusion of placebo (Ringer’s lactate solution) or allogeneic adipose MSCs, intravenously, at three doses. Next, all 32 participants received over a 1-min period one single dose of LPS intravenously (2 ng/kg from *Escherichia coli*, a U.S. standard reference endotoxin, kindly provided by Anthony Suffredini, National Institute of Health, Bethesda, MD, USA) one hour after placebo or MSC infusion. From this randomized study, we selected the eight participants from the placebo arm that received LPS infusion, while saline was infused as a placebo. The samples of these eight participants before and 24 h after LPS infusion and saline infusion were analyzed. The data for non-cholesterol sterols, plasma lipids and lipoprotein concentrations, and CEC are entirely novel and have not been published before. All the participants were apparently healthy young men and had normal medical histories, physical examinations, hematological and biochemical screening values, and electrocardiograms [13]. This study was approved by the Dutch Central Committee on Research involving Human Subjects (CCMO) and the Medical Ethical Committee of the Academic Medical Center (METC CX611-012), Amsterdam (The Netherlands) and was registered at ClinicalTrials.gov as NCT02328612. Written informed consent was obtained from all the participants before the start of this study.

### 2.2. Blood Sampling

Blood samples were collected in EDTA tubes at baseline (T0), as well as 0.5, 1, 2, 3, 4, 5, 6, 7, 8, 9, 10, and 24 h after LPS infusion. Plasma samples were obtained with centrifugation at 1750× *g* for 10 min at 4 °C and stored in small aliquots at −80 °C until use.

### 2.3. Biochemical Analysis

In the samples collected at baseline and after 24 h, plasma TC (CHOD-PAP method; Roche Diagnostic, Mannheim, Germany), HDL-C (CHOD-PAP method; Roche Diagnostic, Mannheim, Germany), and TG concentrations were analyzed enzymatically (GPO-Tinder; Sigma-Aldrich Corp., St. Louis, MO, USA). In these samples, plasma LDL-C concentrations were calculated using the Friedewald equation [22]. HDL functionality, defined as the capacity of radioactive cholesterol efflux from cultured J774 macrophages, using liquid scintillation counting was determined as described elsewhere [23]. Markers for inflammation were analyzed in the plasma samples collected at all time points as described [13].

### 2.4. Non-Cholesterol Sterol and Oxysterol Concentrations

In the samples collected at baseline and after 24 h, plasma non-cholesterol sterol and oxysterol concentrations were analyzed using gas–liquid chromatography–mass spectroscopy (GC-MS) as described before [24,25]. Concentrations of non-cholesterol sterols and oxysterols were standardized for TC concentrations and expressed as μmol/mmol and nmol/mmol cholesterol, respectively. The measured non-cholesterol sterols were campesterol, sitosterol, cholestanol, lathosterol, lanosterol, and desmosterol. TC-standardized sitosterol, campesterol, and cholestanol values were considered as markers for fractional intestinal cholesterol absorption, and TC-standardized lathosterol and desmosterol values as markers for endogenous cholesterol synthesis. The measured oxysterols were 24-OH-, 27-OH, and 7α-OH-cholesterol. TC-standardized 7α-OH- cholesterol and 27-OH-cholesterol values were considered as markers for bile acid formation [20].

### 2.5. Statistical Analyses

The data are presented as means ± SEM in figures and means ± SD in tables. The normality of the data was assessed using the Kolmogorov–Smirnov test. In case of non-normally distributed data, the median and ranges are presented. A paired two-tailed Student’s *t*-test was used to examine differences between lipid and lipoprotein concentrations, HDL functionality, non-cholesterol sterols, and oxysterol and cytokine concentrations at baseline and 24 h after LPS infusion. To evaluate the overall inflammatory responses, the incremental areas under the curves (iAUCs) for 24 h after LPS infusion were analyzed using GraphPad Prism version 9.00 for Windows (GraphPad Software, San Diego, CA). Maximal changes (iMAXs) for a parameter were calculated by subtracting baseline (T0) concentrations from their maximal concentrations. The associations of baseline concentrations as well as changes in non-cholesterol sterol and oxysterol concentrations with the iMAXs or iAUCs of the cytokines were statistically evaluated by calculating the Pearson correlation coefficients. A *p*-value < 0.05 was considered to be statistically significant. All statistical analyses were performed using SPSS 27.0 for Windows (SPSS Inc., Chicago, IL, USA).

## 3. Results

### 3.1. Lipids and Lipoproteins

**Figure 1:**
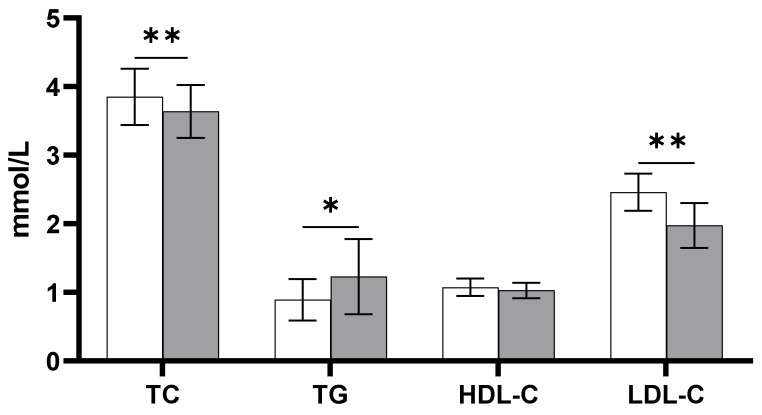
Fasting plasma lipid and lipoprotein concentrations at baseline (white bars) and 24 h following LPS infusion (grey bars) (*n* = 8). Data are presented as means ± SEM. Significantly different from baseline: * *p* < 0.05; ** *p* < 0.01. TC: total cholesterol; TG: triglyceride; HDL-C: high-density lipoprotein cholesterol; LDL-C: low-density lipoprotein cholesterol.

**Figure 2:**
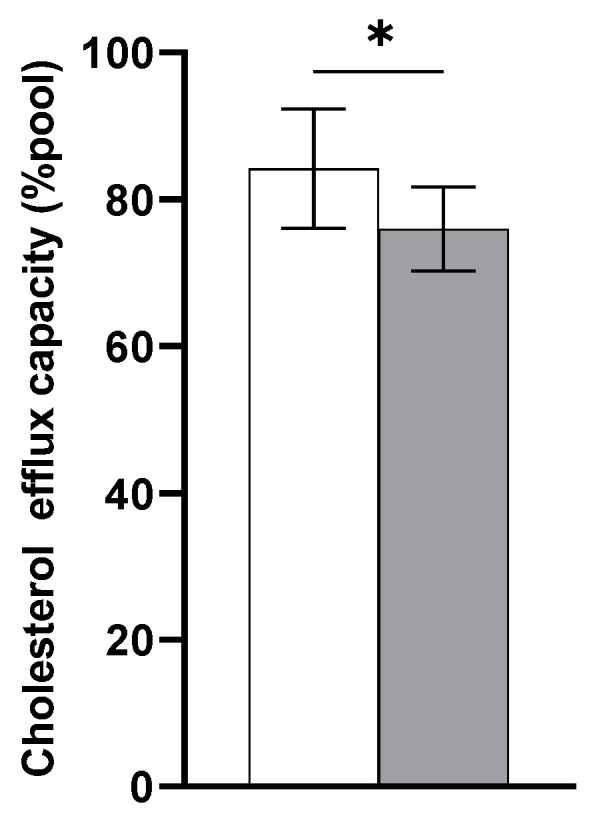
Percentage of cholesterol capacity efflux at baseline (white bar) and 24 h following LPS infusion (grey bar) (*n* = 8). Data are presented as means ± SEM. Significantly different from baseline (* *p* < 0.05). Values are expressed relative to those of a plasma pool of healthy volunteers, which was set at 100%.

Eight healthy Caucasian men with a median age of 23 years (range: 19–25) and a body mass index of 24 kg/m^2^ (22–26) were included in the placebo arm. Plasma TC and LDL-C concentrations were significantly decreased 24 h after LPS infusion ((−0.21 mmol/L; 95% CI: −0.36, −0.05; *p* < 0.05) and (−0.49 mmol/L; 95% CI: −0.72, −0.26; *p* < 0.01), respectively) compared with baseline (Figure 1). However, plasma TG concentrations were significantly increased by 0.34 mmol/L (95% CI: 0.07, 0.61; *p* < 0.05) 24 h following LPS exposure compared with baseline. There were no changes in plasma HDL-C concentrations 24 h after LPS infusion. However, despite unchanged HDL-C concentrations, cholesterol efflux capacity as a measure of HDL functionality was decreased 24 h following LPS infusion compared with baseline (−8.2%; 95% CI: −15.25, −1.26; *p* < 0.05) (Figure 2).

### 3.2. Non-Cholesterol Sterols and Oxysterols

**Figure 3:**
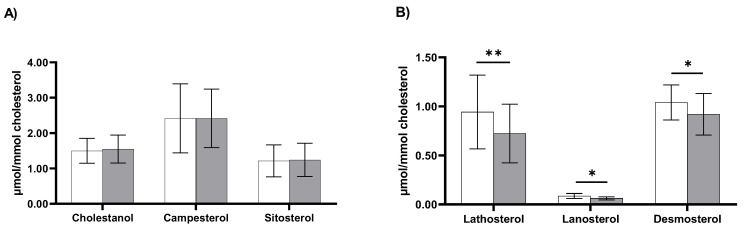
Cholesterol-standardized levels of cholesterol absorption markers (panel (**A**)) and cholesterol synthesis markers (panel (**B**)) at baseline (white bars) and 24 h after LPS infusion (grey bars) (*n* = 8). Data are presented as means ± SEM. Significantly different compared with baseline: * *p* < 0.05; ** *p* < 0.01.

Plasma cholesterol-standardized concentrations of non-cholesterol sterols at baseline and 24 h after LPS infusion are shown in Figure 3. Values for the intestinal cholesterol absorption markers, the TC-standardized levels of campesterol, sitosterol, and cholestanol, were comparable at baseline and 24 h after LPS exposure (Figure 3A). However, TC-standardized levels of the endogenous cholesterol synthesis markers lathosterol, lanosterol, and desmosterol were all significantly lower at 24 h after LPS infusion compared with baseline (Figure 3B). For lathosterol, levels were −0.21 μmol/mmol (95% CI: −0.36, −0.07; *p* < 0.01) lower 24 h after LPS infusion compared with baseline, for lanosterol −0.23 μmol/mmol (95% CI: −0.05, 0.00; *p* < 0.05), and for desmosterol −0.12 μmol/mmol (95% CI: −0.20, −0.03; *p* < 0.05).

**Figure 4:**
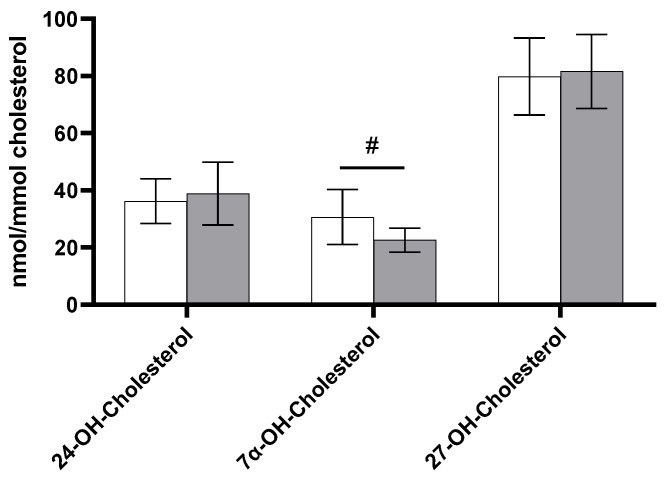
Values at baseline (white bars) and 24 h after LPS infusion (grey bars) for cholesterol-standardized oxysterols (*n* = 8). Data are presented as means ± SEM. ^#^ *p* = 0.050: trend compared with baseline.

As shown in Figure 4, TC-standardized levels of oxysterols were comparable at baseline and 24 h after LPS infusion. For 7α-OH-cholesterol, there was a borderline significant decrease of −8.07 nmol/mmol (95% CI: −16.14, 0.01; *p* = 0.050) 24 h after LPS infusion compared with baseline values. The absolute concentrations of non-cholesterol sterols and oxysterols are shown in Table S1. The results were comparable to those observed for cholesterol-standardized levels.

### 3.3. Inflammatory Responses

The plasma concentrations of a panel of inflammatory markers at baseline and 24 h after LPS exposure as well as the changes are shown in Table 1. Concentrations of the acute phase proteins albumin, C-reactive protein (CRP), and serum amyloid A (SAA) increased 24 h following LPS infusion compared with baseline (all *p* < 0.05). Of the pro-inflammatory cytokines, only tumor necrosis factor (TNFα) concentrations increased 24 h after LPS infusion, whereas IL-8 and IL12p40 concentrations remained unchanged. In addition, concentrations of the anti-inflammatory cytokine IL-10 remained unchanged 24 h after LPS infusion. Myeloperoxidase (MPO) concentrations, an enzyme released upon neutrophil activation, were also increased in response to LPS infusion (*p* < 0.001).

### 3.4. Correlations

We also questioned whether parameters related to cholesterol metabolism at baseline were predictive for the intensity of the LPS-induced systemic inflammatory response. As shown in Table 2, positive correlations were found between baseline TC-standardized desmosterol levels and CRP concentrations at 24 h (r = 0.849; *p* < 0.001) as well as with changes in CRP concentrations (r = 0.829, *p* < 0.05) and iMAX TNFα concentrations (r = 0.917, *p* < 0.05). Moreover, positive correlations were also found between baseline TC-standardized 7α-OH-cholesterol levels and iMAX IL-6 (r = 0.763, *p* < 0.05), iMAX IL-8 (r = 0.766, *p* < 0.05), and iMAX TNFα (r = 0.814, *p* < 0.05) concentrations, and iAUC IL-6 (r = 0.869, *p* < 0.01). Furthermore, positive correlations were found between baseline TC-standardized 27-OH-cholesterol levels and 24 h IL-8 concentrations (r = 0.771, *p* < 0.05) and iAUC TNFα (r = 0.765, *p* < 0.05). Finally, positive correlations were found between baseline TC-standardized cholestanol levels and iMAX MPO (r = 0.758, *p* < 0.05).

For changes, we found that changes in TC-standardized desmosterol and TC-standardized 7α-OH-cholesterol were both negatively correlated with iMAX IL-8 (r = −0.761; *p* < 0.05, and r = −0.856, *p* < 0.01, respectively). Moreover, there were also negative correlations between changes in TC-standardized 7α-OH-cholesterol and iMAX IL-6 (r = −0.751; *p* < 0.05), iMAX TNFα (r = −0.821, *p* < 0.05), and iAUC IL-6 (r = −904, *p* < 0.01).

## 4. Discussion

This study demonstrates that in healthy young male subjects, a transient LPS-induced inflammatory response lowers plasma TC and LDL-C concentrations as well as HDL functionality measured as cholesterol efflux capacity, while plasma TG concentrations are increased. Moreover, endogenous cholesterol synthesis as well as bile acid production were reduced, while intestinal cholesterol absorption did not change. Finally, we found positive correlations between baseline TC-standardized desmosterol and 7α-OH-cholesterol levels with various markers for the inflammatory response and negative correlations between changes in TC-standardized desmosterol and 7α-OH-cholesterol and markers for the inflammatory response. This suggests that an acute LPS-induced transient inflammatory response affects cholesterol metabolism.

Several in vitro, animal, and human studies have already reported possible effects of inflammation on serum lipid and lipoprotein profiles, as well as on the composition, structure, and functionality of HDL particles. Our results on TC and LDL-C concentrations and on TG and HDL-C concentrations are largely in line with the earlier studies. Already in the 1990s, two studies observed induction of hypertriglyceridemia upon LPS, TNF, or IL-1β exposure in rodents [26,27]. In humans, Hudgins et al. demonstrated reductions in serum TC and LDL-C with no effect on HDL-C concentrations in six normal volunteers who were provided with a small dose of endotoxin versus saline [14]. In addition, another study in healthy volunteers, including 10 males and 10 females, reported no changes in serum HDL-C concentrations after LPS infusion [28]. In a more recent study, Zimmetti et al. compared 59 subjects with infections, carcinomas, or autoimmune diseases with 39 controls without infections. Although this study also reported lower serum TC and LDL-C concentrations in patients with inflammation compared with controls, serum TG and HDL-C concentrations were lower [11]. It should, however, be noted that this was a very heterogenous patient population, which could explain the observed differences when compared with our and other studies. During inflammation, the effects on lipoprotein metabolism are not limited to changes in circulating concentrations, but also in HDL particles’ size, structure, and functionality, at least in rodents [7]. In general, inflammation in humans seems to be associated with increases in the HDL component SAA [9,10,28], which is consistent with our observation. Unfortunately, we did not analyze HDL size but did evaluate changes in CEC, which is one of the postulated protective functions of HDLs and is negatively related with CVD development [29]. The decrease in CEC after LPS exposure was in line with other studies in humans upon LPS exposure [15,28,30] and in patients with inflammatory diseases [31,32,33,34].

The question is how these changes in serum lipid and lipoprotein concentrations can be explained. To the best of our knowledge, this is the first study that examined the effects of transient LPS-induced inflammation on plasma markers for intestinal cholesterol absorption, endogenous cholesterol synthesis, and bile acid formation. In one cross-sectional study, no differences in plasma non-cholesterol sterols between subjects with infections, oncologic carcinomas, or autoimmune diseases and controls were reported [11]. However, as already mentioned, the patient population in that study was highly variable, which might have influenced the results. In contrast, we showed that cholesterol synthesis was significantly reduced following LPS infusion, while cholesterol absorption remained unchanged. Our data for cholesterol synthesis is in line with studies in human cell lines but not in animals. For example, adding IL-1 to HepG2 cells inhibited cholesterol synthesis in [35]. However, administering the inflammatory cytokines TNFα, TNFβ, and interferon gamma to mice stimulated hepatic cholesterol synthesis in [36,37,38]. This latter finding was in line with a recent study by Liebergall et al., who reported that proinflammatory stimuli in macrophages from mice upregulated all enzymes involved in cholesterol synthesis, except 24-dehydrocholesterol reductase (DHCR24) [39]. We cannot explain the discrepancies in findings in animals when compared with the in vitro human cell data. In fact, results from animal studies cannot always be extrapolated to humans. Alternatively, it could relate that the way of inducing inflammation was different in all three settings. Regarding the comparability of the serum and plasma samples, we evaluated earlier in our lab if the concentrations of non-cholesterol sterols differ between heparin and EDTA plasma and/or serum samples, and the results showed comparable values for the non-cholesterol sterols between plasma and serum samples (unpublished data).

With respect to bile acid formation, the LPS-induced inflammatory response resulted in decreased bile acid formation, as suggested by a reduction in plasma 7α-OH-cholesterol, which is a precursor in the classic pathway of bile acid synthesis. The 7α-hydroxylase, a rate limiting enzyme in the classical pathway of bile acid synthesis, converts cholesterol into 7α-OH-cholesterol and ultimately, in a series of steps with different enzymes, into bile acids [40]. Two earlier studies in rodents already examined the effects of inflammation on mRNA and protein levels of 7α-hydroxylase after LPS infusion. One study infused Syrian hamsters with LPS, TNFα, or IL-1, while the other study infused rats and mice with LPS. The study in hamsters reported a reduction in the mRNA levels of 7α-hydroxylase [41], whereas the study in rats and mice reported a decrease in the protein levels of 7α-hydroxylase [42]. Both findings are in line with the reduction in 7α-OH-cholesterol that we observed in humans.

In addition to the effects of inflammation on circulating non-cholesterol sterols and oxysterols, it is interesting to note that these sterols also influence inflammation. For example, desmosterol and oxysterols, such as 24S, 25, and 27-OH-cholesterol, have anti-inflammatory properties via activating LXR [43,44,45,46]. Interestingly, these receptors are also known to mediate CEC in vivo and in vitro, and the possible mechanism involves the activity of ABCA1 and ABCG1 [47,48]. In fact, desmosterol has been shown to be the dominant LXR ligand in human atherosclerotic plaques and macrophage foam cells of murine [21], suggesting a reduction in desmosterol is linked with a lower activation of LXR. This might explain the reduction in CEC we observed here after LPS exposure. In a functional way, the reduction in HDL functionality may result in cellular cholesterol accumulation, which may enhance the inflammatory response to remove the infectious agents from the host [49].

Finally, we found unexpected positive associations for baseline TC-standardized desmosterol and 7α-OH-cholesterol levels with the intensity of the inflammatory response. This suggests that higher desmosterol concentrations translate into higher inflammatory responses, which is in contrast with results from a recent study [50]. In that study, the depletion of desmosterol by overexpressing DHCR24 in macrophage foam cells was associated with the activation of inflammatory responses. Moreover, Spann et al. found that activation of macrophage foam cells in the peritoneal cavities of mice was associated with suppression of the hemostatic and anti-inflammatory properties of desmosterol [21]. We can only speculate that these associations are different between animals versus humans, which requires further study.

The present study has some limitations. First, the sample size for this study is small and information about dietary intake is lacking. Second, due to limited sample availability, data for non-cholesterol sterols could only be retrieved at baseline and 24 h after LPS infusion and not in the samples at the timepoints in between, as reported for inflammatory responses. Third, only male subjects were included in the present study, however, there are no indications that non-cholesterol sterols reflecting intestinal cholesterol absorption or endogenous cholesterol synthesis are only valid as markers for these characteristics in male and not in female subjects. The strength of this study is that a transient LPS model was used, which is a highly controlled and reproducible model for studying the effects of a systematic inflammatory responses.

## 5. Conclusions

To conclude, we demonstrated that LPS-induced transient inflammation reduced endogenous cholesterol synthesis and bile acid formation in healthy young men. We speculate that mainly the reduction in cholesterol synthesis explains the observed reductions in plasma TC and LDL-C concentrations. Furthermore, understanding the relation between circulating desmosterol and 7α-OH-cholesterol concentrations at baseline with the intensity of the inflammatory response after LPS exposure warrants further study.

## Figures and Tables

**Table 1:** Plasma concentrations of inflammatory markers before and after 24 h following LPS infusion in all participants (*n* = 8).

		Mean ± SD	*p*-Value
Albumin (g/L)	Baseline	38.43 ± 1.60	
	24 h	40.59 ± 2.32	0.033
	Change	2.16 ± 2.31	
CRP (mg/L)	Baseline	3.01 ± 4.42	
	24 h	24.50 ± 6.83	<0.001
	Change	21.49 ± 2.21	
TNFα (pg/mL)	Baseline	3.02 ± 1.30	
	24 h	3.76 ± 1.37	0.002
	Change	0.74 ± 0.45	
IL-8 (pg/mL)	Baseline	0.32 ± 0.22	
	24 h	0.60 ± 0.46	0.110
	Change	0.29 ± 0.45	
IL-10 (pg/mL)	Baseline	0.19 ± 0.09	
	24 h	0.23 ± 0.13	0.457
	Change	0.04 ± 0.15	
IL12p40 (pg/mL)	Baseline	1.86 ± 1.13	
	24 h	2.25 ± 2.12	0.355
	Change	0.39 ± 1.12	
SAA (mg/L)	Baseline	5.40 ± 1.89	
	24 h	6.64 ± 1.95	0.017
	Change	1.24 ± 1.13	
MPO (ng/mL)	Baseline	2.96± 1.05	
	24 h	5.70 ± 1.56	<0.001
	Change	2.73 ± 1.33	

CRP: C-reactive protein; TNFα: tumor necrosis factor; IL-8: interleukin 8; IL-10: interleukin 10; IL12p40: interleukin 12p40; SAA: serum amyloid A; MPO: myeloperoxidase.

**Table 2:** Correlations between plasma non-cholesterol sterols, oxysterols, and inflammation responses ^‡^.

Variable	Variable	Correlation	*p*-Value
Baseline desmosterol	24 h CRP	0.849	0.008
Baseline desmosterol	∆CRP	0.829	0.011
Baseline 27-OH-cholesterol	24 h IL8	0.771	0.025
Baseline cholestanol	iMAX MPO	0.758	0.029
Baseline desmosterol	iMAX TNFα	0.917	0.010
Baseline 7α-OH-cholesterol	iMAX IL-8	0.766	0.027
Baseline 7α-OH-cholesterol	iMAX IL-6	0.763	0.028
Baseline 7α-OH-cholesterol	iMAX TNFα	0.814	0.049
∆desmosterol	iMAX IL-8	−0.761	0.028
∆7α-OH-cholesterol	iMAX IL-8	−0.856	0.007
∆7α-OH-cholesterol	iMAX IL-6	−0.751	0.032
∆7α-OH-cholesterol	iMAX TNFα	−0.821	0.045
Baseline 27-OH-cholesterol	iAUC-TNFα	0.765	0.027
Baseline 7α-OH-cholesterol	iAUC-IL6	0.869	0.005
∆7α-OH-cholesterol	iAUC-IL6	−0.904	0.002

^‡^ Only significant Pearson coefficients are reported.

## Data Availability

The data presented in this work are fully available without restriction.

## Supplementary Materials

The following supporting information can be downloaded at https://www.mdpi.com/article/10.3390/biomedicines11010126/s1: Table S1: absolute values of non-cholesterol sterol and oxysterols at baseline and 24 h after LPS infusion (*n* = 8); Table S2: cholesterol-standardized levels of non-cholesterol sterol and oxysterols at baseline and 24 h after LPS infusion (*n* = 8); Table S3: fasting plasma lipid and lipoprotein concentrations at baseline and 24 h following LPS infusion (*n* = 8).
