# Supplementary material for: A Transient Inflammatory Response Induced by Lipopolysaccharide Infusion Lowers Markers of Endogenous Cholesterol and Bile Acid Synthesis in Healthy Normocholesterolemic Young Men
Source: Biomedicines. 2023 Jan 4;11(1):126. doi: 10.3390/biomedicines11010126 (PMC9855383; doi:10.3390/biomedicines11010126)
Supplement: Supplementary file 1 [file biomedicines-11-00126-s001.zip › Table S1.pdf]

A transient inflammatory response induced by lipopolysaccharide infusion lowers markers of endogenous cholesterol and bile acid synthesis in healthy normocholesterolemic young men

Mashnafi S[1,2], Baumgartner S [1], Mensink RP[1], Perlee D[3], van Vught LA[3], Lütjohann D[4], Plat J [1*]

[1] Department of Nutrition and Movement Sciences, NUTRIM School of Nutrition and Translational Research in Metabolism, Maastricht University, Maastricht, the Netherlands

[2] Department of Medical Basic Sciences, Faculty of Applied Medical Sciences, AlBaha University, AlBaha, Saudi Arabia

[3] Center of Experimental & Molecular Medicine, Academic Medical Center, University of Amsterdam, The Netherlands

[4] Institute of Clinical Chemistry and Clinical Pharmacology, University Hospital Bonn, Germany

Table S1. Absolute values of non-cholesterol sterol and oxysterols at baseline and 24 h after LPS infusion (n = 8).

Variable	Baseline	24 hours	*p*-value
Cholestanol[#]	5.72 ± 1.16	5.54 ± 1.03	0.631
Lathosterol[#]	3.64 ± 1.52	2.67 ± 1.21	**0.004**
Campesterol[#]	9.23 ± 3.66	8.74 ± 3.11	0.350
Sitosterol[#]	4.68 ± 1.82	4.49 ± 1.73	0.294
Lanosterol[#]	0.32 ± 0.09	0.23 ± 0.07	**0.021**
Desmosterol[#]	4.03 ± 0.93	3.38 ± 1.02	**0.003**
24-OH-cholesterol[*]	139.24 ± 33.27	142.13 ± 46.67	0.839
27-OH-cholesterol[*]	307.18 ± 61.42	296.96 ± 61.02	0.421
7α-OH-cholesterol[*]	117.44 ± 36.43	81.41 ± 13.31	**0.022**

Data are presented as means ± SD. Values are in [#]μmol/L or *nmol/L. Significant differences between baseline and 24 hours samples (paired two-tailed Student's *t*-test) are depicted in bold.
