# Supplementary material for: A Transient Inflammatory Response Induced by Lipopolysaccharide Infusion Lowers Markers of Endogenous Cholesterol and Bile Acid Synthesis in Healthy Normocholesterolemic Young Men
Source: Biomedicines. 2023 Jan 4;11(1):126. doi: 10.3390/biomedicines11010126 (PMC9855383; doi:10.3390/biomedicines11010126)
Supplement: Supplementary file 1 [file biomedicines-11-00126-s001.zip › Table S2.pdf]

A transient inflammatory response induced by lipopolysaccharide infusion lowers markers of endogenous cholesterol and bile acid synthesis in healthy normocholesterolemic young men

Mashnafi S[1,2], Baumgartner S [1], Mensink RP[1], Perlee D[3], van Vught LA[3], Lütjohann D[4], Plat J [1*]

[1] Department of Nutrition and Movement Sciences, NUTRIM School of Nutrition and Translational Research in Metabolism, Maastricht University, Maastricht, the Netherlands

[2] Department of Medical Basic Sciences, Faculty of Applied Medical Sciences, AlBaha University, AlBaha, Saudi Arabia

[3] Center of Experimental & Molecular Medicine, Academic Medical Center, University of Amsterdam, The Netherlands

[4] Institute of Clinical Chemistry and Clinical Pharmacology, University Hospital Bonn, Germany

Table S2. Cholesterol-standardized levels of non-cholesterol sterol and oxysterols at baseline and 24 h after LPS infusion (n = 8).

Variable	Baseline	24 hours	*p*-value
Cholestanol#	1.50 ± 0.12	1.55 ± 0.14	0.601
Lathosterol#	0.94 ± 0.13	0.72 ± 0.10	**0.008**
Campesterol#	2.42 ± 0.34	2.42 ± 0.29	0.991
Sitosterol#	1.21 ± 0.16	1.24 ± 0.17	0.641
Lanosterol#	0.08 ± 0.01	0.06 ± 0.01	**0.046**
Desmosterol#	1.04 ± 0.06	0.92 ± 0.07	**0.015**
24-OH-cholesterol*	36.22 ± 2.77	38.88 ± 3.88	0.480
27-OH-cholesterol*	79.80 ± 4.76	81.61 ± 4.58	0.393
7α-OH-cholesterol*	30.67 ± 3.41	22.59 ± 1.49	0.050

Data are presented as means ± SEM. Values are in #μmol/mmol cholesterol or *nmol/mmol cholesterol. Significant differences between baseline and 24 hours samples (paired two-tailed Student's *t*-test) are depicted in bold.
