# Supplementary material for: A Transient Inflammatory Response Induced by Lipopolysaccharide Infusion Lowers Markers of Endogenous Cholesterol and Bile Acid Synthesis in Healthy Normocholesterolemic Young Men
Source: Biomedicines. 2023 Jan 4;11(1):126. doi: 10.3390/biomedicines11010126 (PMC9855383; doi:10.3390/biomedicines11010126)
Supplement: Supplementary file 1 [file biomedicines-11-00126-s001.zip › Table S3.pdf]

A transient inflammatory response induced by lipopolysaccharide infusion lowers markers of endogenous cholesterol and bile acid synthesis in healthy normocholesterolemic young men

Mashnafi S[1,2], Baumgartner S [1], Mensink RP[1], Perlee D[3], van Vught LA[3], Lütjohann D[4], Plat J [1*]

[1] Department of Nutrition and Movement Sciences, NUTRIM School of Nutrition and Translational Research in Metabolism, Maastricht University, Maastricht, the Netherlands

[2] Department of Medical Basic Sciences, Faculty of Applied Medical Sciences, AlBaha University, AlBaha, Saudi Arabia

[3] Center of Experimental & Molecular Medicine, Academic Medical Center, University of Amsterdam, The Netherlands

[4] Institute of Clinical Chemistry and Clinical Pharmacology, University Hospital Bonn, Germany

Table S3. Fasting plasma lipid and lipoprotein concentrations at baseline and 24 h following LPS infusion (n = 8).

Variable	Baseline	24 hours	*p*-value
Total cholesterol	3.94 ± 0.13	3.56 ± 0.14	**0.005**
Triglyceride	0.89 ± 0.11	1.23 ± 0.19	**0.021**
High-density lipoprotein-cholesterol	1.07 ± 0.04	1.03 ± 0.04	0.152
Low-density lipoprotein-cholesterol	2.46 ± 0.09	1.97 ± 0.11	**0.002**

Data are presented as means ± SEM. Values are in mmol cholesterol. Significant differences between baseline and 24 hours samples (paired two-tailed Student's *t*-test) are depicted in bold.
